# Acid Suppression Therapy Does Not Predispose to *Clostridium difficile* Infection: The Case of the Potential Bias

**DOI:** 10.1371/journal.pone.0110790

**Published:** 2014-10-24

**Authors:** Lena Novack, Slava Kogan, Larisa Gimpelevich, Michael Howell, Abraham Borer, Ciarán P. Kelly, Daniel A. Leffler, Victor Novack

**Affiliations:** 1 Department of Epidemiology, Faculty of Health Sciences, Ben-Gurion University of the Negev, Beer-Sheva, Israel; 2 Clinical Research Center, Soroka University Medical Center, Ben-Gurion University of the Negev, Beer-Sheva, Israel; 3 The University of Chicago Medicine, Chicago, Illinois, United States of America; 4 Infectious Diseases Unit, Soroka University Medical Center, Beer-Sheva, Israel; 5 Division of Gastroenterology, Beth Israel Deaconess Medical Center, Harvard Medical School, Boston, Massachusetts, United States of America; 6 The Celiac Center at BIDMC, Division of Gastroenterology, Beth Israel Deaconess Medical Center, Boston, Massachusetts, United States of America; Charité, Campus Benjamin Franklin, Germany

## Abstract

**Objective:**

An adverse effect of acid-suppression medications on the occurrence of *Clostridium difficile* infection (CDI) has been a common finding of many, but not all studies. We hypothesized that association between acid-suppression medications and CDI is due to the residual confounding in comparison between patients with infection to those without, predominantly from non-tested and less sick subjects. We aimed to evaluate the effect of acid suppression therapy on incidence of *CDI* by comparing patients with CDI to two control groups: not tested patients and patients suspected of having CDI, but with a negative test.

**Methods:**

We conducted a case-control study of adult patients hospitalized in internal medicine department of tertiary teaching hospital between 2005–2010 for at least three days. Controls from each of two groups (negative for CDI and non-tested) were individually matched (1∶1) to cases by primary diagnosis, Charlson comorbidity index, year of hospitalization and gender. Primary outcomes were diagnoses of International Classification of Diseases (ICD-9)–coded CDI occurring 72 hours or more after admission.

**Results:**

Patients with CDI were similar to controls with a negative test, while controls without CDI testing had lower clinical severity. In multivariable analysis, treatment by acid suppression medications was associated with CDI compared to those who were not tested (OR = 1.88, p-value = 0.032). Conversely, use of acid suppression medications in those who tested negative for the infection was not associated with CDI risk as compared to the cases (OR = 0.66; p = 0.059).

**Conclusions:**

These findings suggest that the reported epidemiologic associations between use of acid suppression medications and CDI risk may be spurious. The control group choice has an important impact on the results. Clinical differences between the patients with CDI and those not tested and not suspected of having the infection may explain the different conclusions regarding the acid suppression effect on CDI risk.

## Background

The morbidity and mortality rates caused by *Clostridium difficile* have increased lately, reflecting increased antibiotic use, the aging population and the emergence of high-level resistant strains [1], [2] Outbreaks of CDI have been registered in hospitals worldwide [1], [3], with reports of increased severity of disease, more frequent community acquired disease and rising CDI-associated healthcare costs [4], [5]. The Centers for Disease Control (CDC) have reported that the annual burden of CDI in the US is>350,000 new cases with 14,000 CDI-related deaths. [6]

Antibiotic treatment has been shown to be the main risk factor for development of CDI. [6], [7] Additional, well-established, risk factors include advancing age (e.g. older than 65), hospital admission, severe underlying disease, [8] prolonged hospitalization [9] and invasive gastrointestinal procedures. [10]

During the last decade studies have reported marked overuse of proton pump inhibitors (PPIs). As many as 60% of prescriptions may not follow the criteria of the National Institute for Clinical Excellence, but are administered for non-indicated, prophylactic reasons [11]–[13]. Gastric acid suppression treatment has been shown repetitively to be associated with an increased risk of hospital and community-acquired CDI. [14]–[18]. This association has been explained by the loss of the defensive effect of gastric acid. [12], [19] While this mechanism appears reasonable for vegetative enteric pathogens it is less plausible for CDI where the inoculum is believed to be predominantly in the form of acid-resistant spores. Also, the association between acid suppression therapy and CDI has not been universal and was not found in some studies. [12], [20]

One of the major limitations of these pharmaco-epidemiological studies is a potential bias inherently associated with this type of analysis: despite the multivariate adjustment the two comparison groups (with and without acid suppression) might differ significantly. Patients who develop CDI are known to be more ill than most other hospital patients. Thus they may be more likely to carry risk factors and exposures that lead to the use of acid suppression therapy. Put differently, the epidemiologic association may result from severe underlying disease being associated with CDI and, in parallel, leading to increased PPI use.

We hypothesize that the comparison groups used to examine the association between acid suppression therapy and CDI are intrinsically unsuited due to their very different clinical characteristics leading to bias. Therefore, to address this concern, we conducted a nested case-control study of CDI patients with two separate matched control groups: one with suspected CDI but negative stool testing and a second without suspected CDI.

## Methods

### Study Population and Study Groups Definition

The study population comprised adult patients hospitalized in internal medicine wards of Soroka University Medical Center (SUMC) during the period 2005–2010. SUMC is a 1100-bed tertiary teaching hospital and the only provider of in-hospital care for the population of 700,000 in southern Israel. Patients included in the study had to spend at least 3 days in the hospital and cases had to be hospitalized 3 days before infection was detected (to qualify as a hospital-acquired infection). We chose not to include patients hospitalized after 2010 in light of growing awareness in our hospital of the reported risk of CCDI associated with use of acid-suppressor medications, supported by the FDA safety announcement issued in Feb 2, 2012 [21].

Cases were defined as a first incidence of a positive stool *C. difficile* toxin (by enzyme-linked immunosorbent assay) in a patient with diarrhea. For each case we matched one control with a negative C. difficile toxin test and one who was not suspected to have CDI and was not tested. Both types of controls were matched by gender, age (with a caliper of 5 years) and hospitalization within 12 months before or after the date of the case hospitalization. Controls with a negative test for CDI were also matched by the primary diagnosis.

### Definitions

Treatment by acid-suppressors was defined as use of a H_2_ receptor antagonist (H_2_RA) or PPI three months prior to admission and during the index hospitalization. The H_2_RA medications included famotidine and ranitidine; PPI medications included omeprazole, lansoprazole and pantoprazole. We further defined exposure to acid suppression by three levels of intensity: (a) patients not receiving acid-suppressor medications, (b) patients receiving only H_2_RA medications, and (c) patients receiving one daily dose of PPI treatment.

Antibiotic treatment was defined by the extent of exposure to antibiotics and was analyzed in 3 groups by the expected risk of CDI: i.e. no antibiotics administered, low-risk and high- intermediate risk antimicrobials. The group of high-intermediate risk antibiotics included fluoroquinolones, cephalosporins, beta-lactams, macrolides, carbapenems, sulfonomides and clindamycin. All other types of antibiotics were included in the low-risk category. This breakdown of antibiotics medications follow the definitions set in our previous investigation [22], as well as by UpToDate web site [23].

Primary diagnosis and co-morbidities were defined by the International Classification of Diseases, 9th Revision (ICD-9). We used the Charlson index to compute the burden of co-morbid conditions. The overall comorbidity score reflects the cumulative increased likelihood of one-year mortality; the higher the score, the more severe the burden of comorbidities. [24]

No informed consent was required, as the current research was based only on patients' medical records collected from the hospital admission-discharge-transfer (ATD) database. Patients' records were anonymized and de-identified prior to analysis. This approach, as well as the protocol of the study, has been approved by the Soroka University Medical Center IRB committee.

### Statistical Analysis

Continuous variables were presented as means±standard deviation (sd), median, minimum and maximum; they were compared between study groups using Wilcoxon and t-test, depending on the variable distribution. Categorical variables were presented as percent out of available cases and compared between study groups using univariate conditional logistic regression technique. Cases were compared separately to each type of control. Multivariate analysis for identifying independent factors affecting CDI was performed using conditional logistic regression. We restricted the study population in the multivariable analysis to those cases with matched controls to keep the comparisons balanced. We performed sensitivity analysis on a sub-set of the study population, where each case was matched to both types of controls.

## Results

### Overview

**Figure 1:**
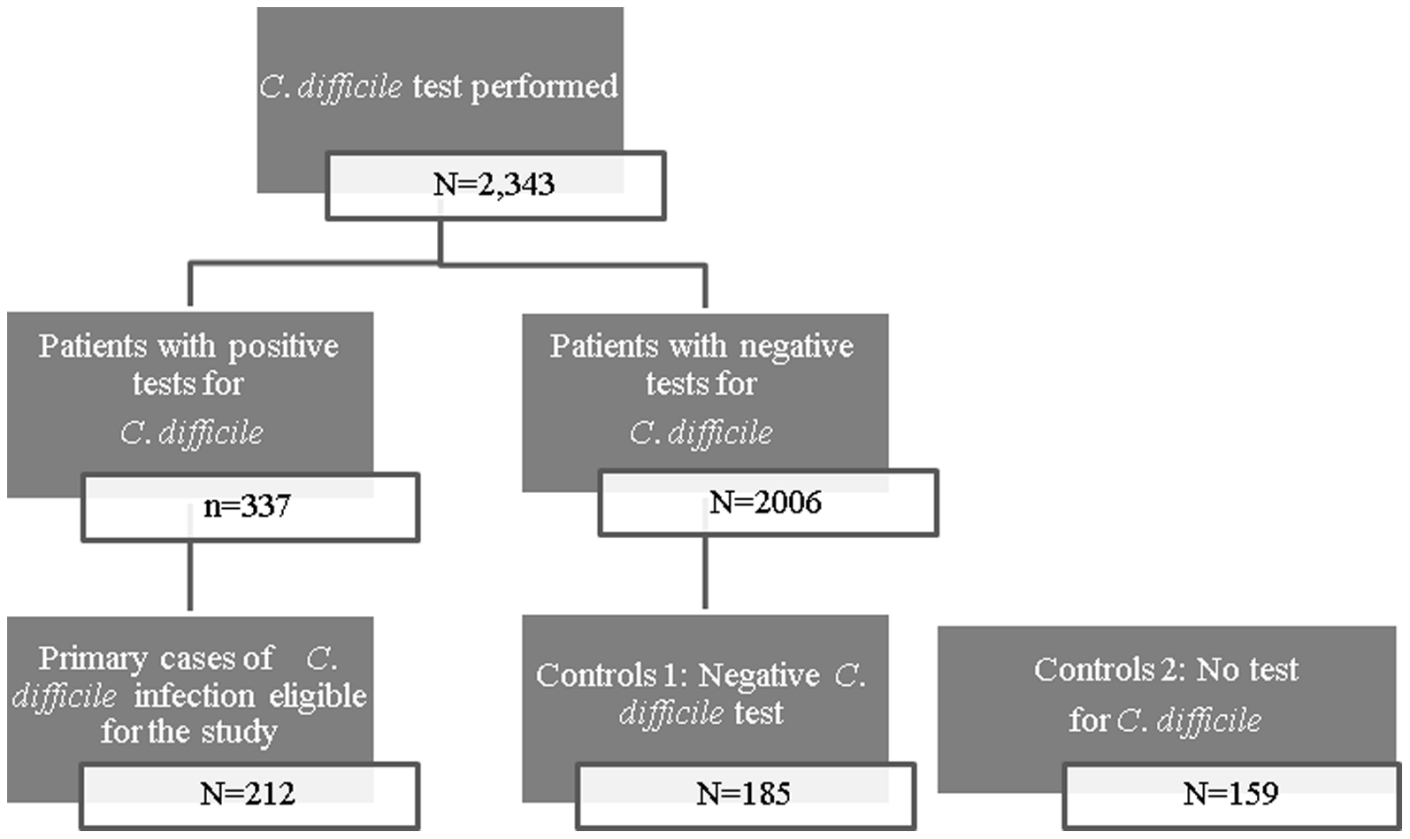
Flowchart of enrolled patients.

During the study period (2005–2010) the SUMC laboratory tested 2,343 stool samples for CDI, 337 were positive (Figure 1) and 212 out of 337 were found eligible for the study (main reasons for non-eligibility were: hospital stay shorter than 72 hours, age below 18, not primary case of CDI). There were 185 patients with a negative stool test and 159 patients without a test who could be matched to the cases. Both types of controls were matched only to 132 cases.

### The study population

Approximately half of the patients were women (51.4%) and on average 69 years old (Table 1). As matching by age was performed with a caliper of 5 years, there was a small discrepancy in age between the study groups; controls with a negative test result were one year older and controls without a test were 2 years older compared to their cases. Even though being clinically marginally important, the two-years difference in age between cases and the controls without a test was statistically significant (p-value<0.001). Of note, 19.3% of the CDI patients lived in nursing homes, compared to 10.8% in the 2 groups of controls (p-value = 0.042). Treatment by antibiotics was more intense within the CDI group than in patients without CDI (Table 2).

**Table 1:** Demographic Characteristics of the Study Population.

Demographic characteristics	Cases: Patients testing positive for *C. difficile* (N = 212)	Patients without *C. difficile* infection	
		Patients negative on *C. difficile* test - Controls type 1 (N = 185)	Patients without a *C. difficile* test - Controls type 2 (N = 159)
**Age, years**			
Mean±SD	68.2±16. 9	69.0±16.9	71.2±17.5
Median	72.0	74.0	77.0
Min; Max	18.0–92.0	18.0–92.0	22.0–97.0
(pv vs. cases)		(pv = 0.084)	(pv<0.001)
**Female Gender, % (n/N)**	53.3% (112)	55.7% (103)	51.6% (82)
(pv vs. cases)		(pv = 1.000)	(pv = 1.000)
**Family status, %(n/N)**			
Married	62.5%(130/208)	58.3%(98/168)	58.5%(93)
Divorced	3.4%(7/208)	5.4%(9/168)	3.8%(6)
Widow	9.1%(19/208)	7.7%(13/168)	12.6%(20)
Not Married	7.2%(15/208)	7.1%(12/168)	7.5%(12)
(pv vs. cases)		(pv = 1.000)	(pv = 0.330)
**Country of birth, %(n/N)**			
Israel	21.2%(45)	19.5%(36)	20.8%(33)
Former USSR	25.0%(53)	25.4%(47)	30.2%(48)
Asia	33.5%(71)	28.6%(53)	23.9%(38)
Europe	7.5%(16)	9.2%(17)	19.5%(31)
North America	4.7%(10)	4.3%(8)	1.9%(3)
Africa	4.7%(10)	2.7%(5)	3.1%(5)
(pv vs. cases)		(pv = 0.143)	(pv = 0.596)
**Residence %(n/N)**			
Home	78.3%(166)	86.5%(160)	86.8%(138)
Nursing Home	19.3%(41)	10.3%(19)	11.3%(18)
Other	2.4%(5)	3.2%(7)	1.9%(3)
(pv vs. cases)		(pv = 0.087)	(pv = 0.143)
**Type of Residency, %(n/N)**			
City	79.4%(167)	75.8%(138)	85.8%(133)
Non-urban settlement	7.1%(15)	7.0%(13)	7.0%(11)
Kibbutz	3.8%(8)	1.6%(3)	3.9%(6)
Bedouin-Arab village	8.1%(17)	6.6%(12)	4.5%(7)
(pv vs. cases)		(pv = 0.132)	(pv = 0.067)

**Table 2:** Clinical Characteristics of the study population during hospitalization, by study groups.

Clinical Characteristics	Cases: Patients testing positive for *C. difficile* (N = 212)	Patients without *C. difficile* infection	
		Patients negative on *C. difficile* test - Controls type 1 (N = 185)	Patients without a *C. difficile* test - Controls type 2 (N = 159)
**Procedure during Hospitalization, % (n)** *(pv vs. cases)*			
Colonoscopy	6.1% (13)	7.1%(13) (pv = 1.000)	2.5%(4) (pv = 0.092)
Surgery	22.9% (47/205)	28.2%(51/181) (pv = 0.590)	3.8%(6) (pv<0.001)
**Feeding, %(n)**			
Oral	71.2% (151)	67.6%(125)	85.6%(136)
Nasogastric tube	25.5% (54)	31.9%(59)	12.6%(20)
PEG	3.3% (7)	0.5%(1)	1.9%(3)
*(pv vs. cases)*		(pv = 0.925)	(pv = 0.003)
**Antibiotics 3 months prior to or during hospitalization, % (n)** *(pv vs. cases)*	96.7% (205)	91.9% (170) (pv = 0.064)	66.0% (105) (pv<0.001)
Before Hospitalization	45.8% (97)	20.0% (37) (pv<0.001)	19.5% (31) (pv<0.001)
During Hospitalization	93.4% (198)	88.1% (163) (pv = 0.151)	64.2% (102)(pv<0.001)
**WBC**			
Mean±SD	15.7±18.6	11.9±7.9	11.6±7. 7
**Positive result obtained for test in, % (n)** *(pv vs. cases)*			
Bacteriology	54.7%(116)	48.1%(89) (pv = 0.271)	25.3%(40) (pv<0.001)
Blood	19.3% (41)	23.8% (44) (pv = 0.499)	9.4% (15) (pv = 0.002)
Sputum	9.1% (19)	11.4%(21) (pv = 0.398)	0.6% (1) (pv<0.001)
Urine	40.6%(86)	27.5%(51) (pv = 0.014)	14.5%(23) (pv<0.001)
**Length of stay, days**			
Median	16.0	13.0	5.0
Min-Max	0.0–440.0	1.0–127.0 (pv = 0.078)	1.0–69.0 (pv<0.001)
**Mortality, %(n)** *(pv vs. cases)*			
In-hospital	21.2% (45)	18.4%(34) (pv = 0.409)	13.2%(21) (pv = 0.010)
At 1 year follow-up	53.3%(113)	45.4%(84) (pv = 0.110)	37.7%(60) (pv<0.001)

Distribution of primary diagnoses is shown in table 3, and was not different between the study groups. The majority of patients were hospitalized due to an infectious disease (62.8%), followed by 17.3% with a diagnosis of a cardiovascular disorder and 9.4% patients hospitalized due to a neoplasm. The group of CDI cases was similar to the group of controls with a negative *C. difficile* test by their co-morbidities and Charlson score (Table 3). Controls without a test had significantly lower rates of pneumonia and anemia (p-value = 0.030 and p-value<0.001, respectively). The proportion of patients fed by nasogastric tube was similar in CDI cases (25.5%) and in controls with a negative test (31.9%) but was lower in control patients without a test (12.6%). To summarize, patients with CDI were similar to controls with a negative test, while controls without a suspicion for CDI had lower clinical severity in several respects. These clinical differences were reflected in a longer hospitalization and higher mortality rate within the patients with a test compared to the patients without (median length of stay was 15 days and 1-year mortality rate reached 49.6% in the group with the test (controls and cases), compared to 5 days of stay (p-value<0.001) and mortality rate 37.7% (p-value = 0.11) in the group without the test.

**Table 3:** Clinical Characteristics of the study population at admission, by study groups.

Clinical Characteristics	Cases: Patients testing positive for *C. difficile* (N = 212)	Patients without *C. difficile* infection	
		Patients negative on *C. difficile* test - Controls type 1 (N = 185)	Patients without a *C. difficile* test - Controls type 2 (N = 159)
**Primary Diagnosis, % (n)**			
Cardiovascular	16.5% (34)	17.8% (33)	18.2% (29)
Infectious	67.0% (142)	58.9% (109)	61.6% (98)
Neoplasm	8.0% (17)	11.4% (21)	8.8% (14)
Orthopedics	3.3% (7)	2.7% (5)	0.6% (1)
Renal	2.8 (6)	2.7% (5)	6.9 (11)
Others	2.8 (6)	6.5% (12)	3.8 (6)
*(pv vs. cases)*		(pv = 0.370)	(pv = 0.199)
**Co-morbidities, % (n)** *(pv vs. cases)*			
History MI	19.8%(42)	27.6%(51) (pv = 0.182)	25.2%(40) (pv = 0.532)
Heart failure	43.3%(94)	40.5%(75) (pv = 0.177)	40.3%(64) (pv = 0.306)
Chronic Pulmonary Disease	8.0%(17)	8.6%(16) (pv = 0.839)	11.3%(18) (pv = 0.850)
Pneumonia	30.2% (17)	30.3% (56) (pv = 1.000)	20.8% (33) (pv = 0.030)
Diabetes	34.4%(73)	37.3%(69) (pv = 1.000)	34.6%(55) (pv = 0.810)
Hypertension	46.2%(98)	48.1%(89) (pv = 0.644)	52.2%(83) (pv = 0.550)
Chronic Renal Failure	13.7%(29)	14.1%(26) (pv = 0.880)	13.2%(21) (pv = 0.424)
Peptic Ulcer Disease	4.7%(10)	4.9%(9) (pv = 1.000)	2.5%(4) (pv = 0.344)
Cancer	19.3%(41)	26.5%(49) (pv = 0.057)	19.5%(31) (pv = 1.000)
Anemia	40.1%(85)	37.8%(70) (pv = 1.000)	28.3%(43) (pv = 0.001)
**Charlson Index**			
Mean±SD	5.0±3.3	5.6±3.9	5.0±3.3
Median	5.0	6.0	5.0
Min-Max *(pv vs. cases)*	0.0–19.0	0.0–18.0 (pv = 0.114)	0.0–15.0 (pv = 0.520)

### Acid suppression therapy

Treatment by acid-suppression medication prior to hospitalization was recorded in 38% patients, without differences between the study groups (table 4). However, as we noticed previously with clinical characteristics, the frequency of treatment by acid suppression medications was similar between cases and controls with a negative test but different from the controls without a test. Administering of H_2_RA was more common within patients with a *C. difficile* test, 21.7% and 20.0% for those with a positive or negative result, respectively, compared to 13.8% for those with no test. In other words, CDI cases were not different from the controls suspected for having CDI (and thus tested) in terms of their exposure to H_2_RA (p-value = 0.511), but their exposure to H_2_RA was higher compared to the controls without a test (at borderline significance level of 0.074). Of note, exposure to PPI therapy was more frequent within those who tested negative for CDI than in the group of CDI cases (48.6% vs. 36.8%, p-value = 0.004).

**Table 4:** Exposure to Acid-suppression medications, by study groups.

Acid suppression medication	Cases: Patients testing positive for *C. difficile* (N = 212)	Patients without *C. difficile* infection	
		Patients negative on *C. difficile* test - Controls type 1 (N = 185)	Patients without a *C. difficile* test - Controls type 2 (N = 159)
**Acid suppression % (n/N)**			
*Within 3 months prior to hospitalization*			
H_2_RA	8.5% (18)	11.4% (21) (pv = 0.275)	6.9% (11) (pv = 0.283)
PPI	26.4% (56)	31.3% (58) (pv = 0.223)	29.6% (47)(pv = 0.892)
**Acid suppression % (n/N)**			
*During hospitalization*	58.5% (124)	68.7% (127) (pv = 0.041)	47.2% (75) (pv = 0.008)
*By type of medication* (pv vs. cases)			
H_2_RA	21.7% (46)	20.0%(37) (pv = 0.511)	13.8%(22) (pv = 0.074)
PPI	36.8% (78)	48.6%(90) (pv = 0.004)	33.3%(53) (pv = 0.222)

Results of a multivariable analysis of factors associated with the *C.Difficile* infection are presented in table 5. After adjusting for well-established risk factors, i.e. Charlson index, residence in a nursing home and treatment by antibiotics, treatment by acid suppression medications showed a protective trend on risk for CDI (OR = 0.66; p-value = 0.059) within the group of patients suspected for infection (i.e. tested for CDI). However, as reported by other studies, treatment with these medications was associated with an increased likelihood of the infection when CDI cases were compared to the controls that were not tested (OR = 1.88, p-value = 0.032). Similar trends were obtained for the effect of H_2_RA and PPI medications, when tested separately. Treatment by H_2_RA showed no risk increase whereas the administration of a PPI significantly reduced the likelihood of CDI within the patients who were tested (OR = 0.54; p-value = 0.019). Each medication was associated with an increased likelihood of CDI when CDI cases were compared to untested controls, however with p-values approaching significance (OR_H2RA_ = 1.99, p-value = 0.075 and OR_PPI_ = 1.82, p-value = 0.077).

**Table 5:** Factors affecting *C. difficile* infection, results of a multivariate analysis by conditional logistic regression.

Patients' Characteristics^1^	Patients negative on *C. difficile* test - Controls type 1 (N = 185)		Patients without a *C. difficile* test - Controls type 2 (N = 159)	
	Odds Ratio (CI 95%)	p-value	Odds Ratio (CI 95%)	p-value
Acid suppression during hospitalization	0.66 (0.44–1.02)	0.059	1.88 (1.06–3.36)	0.032
Antibiotic Therapy (before and during hospitalization)	3.03 (1.07–8.62)	0.037	12.97 (4.59–36.61)	<0.001
Residing in a nursing home	1.98 (1.07–3. 669)	0.029	2.59 (1.09–6.18)	0.031

1 The model included adjustment to Charlson Index.

### Sensitivity and Missing Data Analysis

Not all 212 CDI cases were matched to both types of controls. Sensitivity analysis on a fully matched set of 132 cases with both controls available showed no difference in study conclusions.

Furthermore, 27 CDI cases had no tested negative controls and 53 CDI had no controls from the group without a test. Closer inspection of distribution of exposure within “not-paired” CDI cases reveal that 27 cases without negative tested controls to the rest of the sample used in the analysis. However, 53 cases without a match in non-suspected group had lower exposure to acid-suppression therapy (47% in non-paired cases vs. 62% in the paired cases used in the main analysis). We have simulated a univariable conditional logistic regression analysis on 1000 samples assuming inclusion of the group of unpaired cases, which would have resulted in overall 58.2% of exposure in all cases. The analysis showed that the risk estimate would have decreased from 1.88 (table 4) to 1.60, and would have maintained the statistical significance.

## Discussion

In this study we hypothesized that the comparison of CDI cases to all hospital patients without CDI, as adopted in a majority of studies, brings bias to the results based on the overall severity of illness in the CDI patients. Severely ill patients may be more likely to receive acid suppression medications leading to an apparent association of these medications with CDI. We assessed our hypothesis in a case-control setting, in which patients with a positive test for *C. difficile* (CDI cases) were compared separately to patients suspected of CDI but with a negative test (a group that may be clinically more similar to CDI patients) and controls in whom CDI was not suspected or tested for.

The data obtained in our study brought us to a different understanding of the association between acid-suppression medications and CDI. From the comparison of demographical, clinical and procedural characteristics of the study groups – it became apparent that the group of cases is similar to the group of controls with suspected CDI but having a negative test. Furthermore, both are very different from the patients without a test. The CDI cases and the controls with a negative test are united by a clinical suspicion for having CDI (and therefore being tested for it). This clinical suspicion identifies a group of subjects with high underlying disease burdens and high subsequent death rates. The similarity between CDI cases and controls with a negative test for CDI and their difference from patients not suspected of CDI is statistically evident for the diagnoses of pneumonia and/or anemia, more prevalent co-morbidities, as well as more frequent procedures compared to patients in whom the test was not performed. The patients suspected for CDI also more frequently required nasogastric feeding and more frequently had a positive bacterial culture in blood, sputum or urine, compared to controls without a CDI test. These markers of increased underlying disease severity were also associated with adverse clinical outcomes including longer hospitalizations and higher mortality rates.

Prior exposures to acid-suppressing medications in the community was not different between the study groups, but changed following hospitalization, when patients not suspected of having CDI were less likely to receive acid-suppressing medication compared to those with suspected or proven CDI. This discrepancy resulted in an estimated independent adverse effect for exposure to H_2_RA and PPI medications, with OR = 1.88 (p-value = 0.032) for those with CDI compared to the controls that were not tested. Quite remarkably, the patients with a negative *C. difficile* test were more likely to be exposed to intensive acid suppression treatment (48.6% exposed to PPI) compared to those with CDI (36.7% exposed to PPI). In keeping with this finding, the multivariable analysis did not show a detrimental effect of acid suppression medications (OR = 0. 66, p-value = 0.059) in comparison between patients with CDI and those with suspected CDI. Based on the contradictory findings we cannot conclude that there is an adverse effect of exposure to acid-suppressing medication and CDI risk.

Acid anti-secretory medications and particularly PPIs may lead to diarrhea. [25] Thus, patients taking PPIs may be more likely to be tested for CDI but be negative, as most of the patients would have PPI-induced diarrhea, in comparison to general population of patients with more prevalent CDI-induced diarrhea. To illustrate this let us consider the following hypothetical example, where we have 100 patients with diarrhea and no PPI treatment with CDI prevalence of 5% (5 cases); and let us assume that PPI treatment is associated with 2 folds increase in diarrhea risk, leading to a situation where in the same hospital population, but now treated with PPI we will have twice as many patients with diarrhea – 200. Assuming that PPI treatment does not increase CDI risk, among these 200 subjects we will still have the same 5 cases of CDI (2.5%). We might conclude that statistically speaking PPI treatment is associated with a protective effect against CDI – a decrease from 5% to 2.5% incidence. This may, in part, account for the protective, but spurious PPI effect observed in our study.

Our findings disagree directly with the widely held opinion that acid suppression medications are a risk factor for CDI. This discrepancy inevitably leads to questions regarding the validity of our methodology. For example, possible inaccuracies of the *C. difficile* tests might have concealed the effect. However, the assessed effect is in fact significantly opposite, which cannot be fully explained by laboratory error. Our results showing an adverse effect of acid-suppression medications in comparison with controls not tested for CDI, is in fact, consistent with the published research by Pohl [18], Howell et al [22], Bavishi and Dupont [26] or by Pakyz et al [27], where the control group consisted of all patients not-positive for CDI. In those studies the group of patients with a negative CDI test was diluted by the majority who were not tested. Therefore, the published data parallels the results in our study whereby untested controls show reduced use of acid suppression medications compared to those with CDI. Of note, few studies comparing cases to patients with negative CDI results, e.g. by Shah et al in 2000 [28] or McFarland et al in 2007 [29], found no association between exposure to acid-suppression and the infection.

Previous research [30] and our data support the hypothesis that the administration of acid-suppressors and testing for CDI (whether positive or negative) are both markers of an unmeasured severity of illness. Comparing cases and controls within a group of patients with the test, who are similar in their degrees of disease severity, could produce the level of adjustment needed to unmeasured confounders. A cohort strategy might lack this necessary level of adjustment and result in biased estimates.

Our analyses confirmed several well-established risk factors for the development of CDI including antibiotic therapy and residence in a nursing home. Conversely, in our study acid suppression therapy was not associated with an increased risk for CDI when patients with similar levels of disease severity were compared. Acid suppression therapy and CDI both track patients with more severe disease leading to an apparent association that does not appear to be primary or causative. Furthermore, these results draw into question the recent Drug Safety Labeling Changes made by the US FDA warning about an association between PPI use and risk for CDI as well as similar warnings in current CDI treatment guidelines. [31]

The study findings, however, have to be treated with caution in view of its limitations. Not all CDI cases were matched to both types of controls; however sensitivity analysis on a fully matched set (132 cases + 2 controls) did not changed conclusions from the main analysis. Likewise, sensitivity analysis of cases without negatively tested controls (27 cases) and cases without non-suspected controls (53 cases) resulted in the same statistical inference. These findings support the internal validity of the study.

We assume that some of the questionable findings in the study may have roots in a possible bias, e.g. the spurious protective effect of PPIs, as discussed previously. The positive effect of PPIs use could have been inflicted by a synergistic interaction that becomes prominent when antibiotics and PPIs are used together [32]. However, the interaction should not affect our findings since the rates of the antibiotics exposure were similar in tested controls (88.1%) and cases (93.4%).

The results might be not fully generalizable, as the study was conducted in a single hospital. On the other hand, the point estimates obtained throughout the analysis is within the range of the effect reported in other studies, which supports the external validity of the results.

To conclude, the potential study bias described in the current analysis needs to be addressed in future research by a careful selection of the relevant control groups for CDI patients.
